# Diabetic microvascular disease in non-classical beds: the hidden impact beyond the retina, the kidney, and the peripheral nerves

**DOI:** 10.1186/s12933-023-02056-3

**Published:** 2023-11-15

**Authors:** Dídac Mauricio, Mònica Gratacòs, Josep Franch-Nadal

**Affiliations:** 1 DAP-Cat group, Unitat de Suport a la Recerca Barcelona, Fundació Institut Universitari per a la recerca a l’Atenció Primària de Salut Jordi Gol i Gurina (IDIAPJGol), Barcelona, Spain; 2 CIBER of Diabetes and Associated Metabolic Diseases (CIBERDEM), Instituto de Salud Carlos III (ISCIII), Barcelona, Spain; 3 Department of Endocrinology and Nutrition, Hospital de la Santa Creu i Sant Pau, IR Sant Pau, Barcelona, Spain; 4 Department of Medicine, University of Vic - Central University of Catalonia, Vic, Spain

**Keywords:** Diabetes mellitus, Diabetes complications, Microvessels, Microcirculation, Diabetic vascular complications, Diabetic microangiopathy, Microvascular complications, Microvascular changes

## Abstract

Diabetes microangiopathy, a hallmark complication of diabetes, is characterised by structural and functional abnormalities within the intricate network of microvessels beyond well-known and documented target organs, i.e., the retina, kidney, and peripheral nerves. Indeed, an intact microvascular bed is crucial for preserving each organ’s specific functions and achieving physiological balance to meet their respective metabolic demands. Therefore, diabetes-related microvascular dysfunction leads to widespread multiorgan consequences in still-overlooked non-traditional target organs such as the brain, the lung, the bone tissue, the skin, the arterial wall, the heart, or the musculoskeletal system. All these organs are vulnerable to the physiopathological mechanisms that cause microvascular damage in diabetes (i.e., hyperglycaemia-induced oxidative stress, inflammation, and endothelial dysfunction) and collectively contribute to abnormalities in the microvessels’ structure and function, compromising blood flow and tissue perfusion. However, the microcirculatory networks differ between organs due to variations in haemodynamic, vascular architecture, and affected cells, resulting in a spectrum of clinical presentations. The aim of this review is to focus on the multifaceted nature of microvascular impairment in diabetes through available evidence of specific consequences in often overlooked organs. A better understanding of diabetes microangiopathy in non-target organs provides a broader perspective on the systemic nature of the disease, underscoring the importance of recognising the comprehensive range of complications beyond the classic target sites.

## Introduction

### Microcirculation: structure and physiological functions

Microcirculation refers to the ubiquitously distributed and interconnected network of the most distant blood vessels with diameters ranging from 5 to 100 μm, encompassing arterioles, capillaries, and venules [1, 2]. Capillaries are composed of endothelial cells (ECs) surrounded by pericytes and a basement membrane (BM), whereas arterioles have an additional thick layer of smooth muscle cells (media) to withstand blood pressure [3].

The complex microcirculation system works as a functional unit controlling several critical physiological processes [2, 4]: facilitating oxygen exchange between blood and tissues along a concentration gradient; regulating microvascular flow and intravascular pressure within individual organs by modulating the luminal diameter of arterioles to meet tissue perfusion with oxygen demand; transporting nutrients and hormones through passive diffusion or transcytosis; leading transcapillary water movement by balancing hydrostatic capillary and osmotic interstitial fluid pressures; regulating solute exchange, including small hydrophilic solutes (e.g., ions, sugars, certain drugs) and macromolecules (proteins, carbohydrates, lipids); and coordinating localised responses to inflammation by enabling rapid and abundant leukocyte transmigration from blood to tissue across ECs.

Endothelial cells constitute a significant proportion of the lining in the circulatory system’s microvasculature [3]. By acting as a selective barrier between the blood and the surrounding tissues, any impairment or dysfunction of ECs plays a pivotal role in developing, progressing, or exacerbating the diverse microvascular dysfunctions that affect different organ systems and their respective physiological processes.

### Mechanisms of diabetic microvascular damage

Intensive blood glucose control has been shown to effectively prevent the onset or delay the progression of diabetic microvascular complications, while its effect is modest or even neutral on the prevention of macrovascular disease [5]. This finding has led to the prevailing assumption that intracellular hyperglycaemia is closely associated with microvascular complications in diabetes mellitus (DM).

Both chronic/intermittent hyperglycaemia and glycation processes contribute to the overproduction of reactive oxygen species (ROS) by mitochondria of the ECs, triggering the activation of several damaging pathways [6–12]: increased glucose and other sugars efflux through the polyol pathway; overactivity of the hexosamine pathway; activation of the protein kinase (PKC) pathway; and an overload of the glycation pathway (dicarbonyl stress). The latter leads to the formation of highly reactive carbonyl species, primarily methylglyoxal, which trigger non-enzymatic glycation and also result in the irreversible accumulation of advanced glycation end products (AGE) and increased expression of AGE receptor (RAGE) and its activating ligands such as macrophages, vascular endothelial cells, and vascular smooth muscle cells.

The aforementioned hyperglycaemia-induced unbalanced biochemical pathways lead to the development and progression of diabetic microangiopathy through different adverse structural and functional changes: ECs injury in vessels between primary arterioles and venules, resulting in reduced blood flow and increased permeability [13]; thickening of BMs, hindering the normal supply of oxygen and nutrients to cells [14, 15]; induction of a pro-inflammatory state, leading to chronic inflammation, further damaging the endothelium [16]; coexistence of pro- and anti-neovascularization responses (angiogenesis paradox) [17], which causes uncontrolled formation of premature blood vessels in some tissues like the retina and deficiency in the formation of small blood vessels in peripheral tissues like the skin, impairing tissue repair and regeneration (e.g., impaired wound healing); and induction of a pro-thrombotic state due to increased procoagulant activity, leading to microthrombosis and subsequent tissue ischemia and damage [18]. Insulin resistance, in addition to hyperglycaemia, has been involved in microvascular damage by decreasing nitric oxide (NO) production. This reduction activates the ras–mitogen-activated protein kinase (MAPK), leading to endothelial dysfunction, as well as stimulating vascular smooth muscle cell (SVMC) proliferation and migration [19].

### Targets of diabetic microvascular injury

While most cell types can effectively reduce glucose transport when exposed to extracellular hyperglycaemia and maintain a constant glucose concentration, ECs are particularly vulnerable to intracellular hyperglycaemia-induced damage due to their limited ability to regulate transport rates [10, 20]. For instance, adipocytes and muscle cells predominantly express GLUT4, a glucose transporter that increases cellular glucose uptake for storage as glycogen in response to insulin, thereby contributing to a rapid reduction in blood glucose levels [21]. In contrast, ECs primarily express GLUT1, which is unresponsive to insulin, and notably, they do not express GLUT4 [22]. This allows ECs to transport glucose directly from the bloodstream, independently of insulin levels, and their glucose uptake closely mirrors blood glucose concentrations [22]. Of note, GLUT-1 serves as the primary glucose transporter in cells that form various blood-tissue barriers, such as those composing the blood-brain barrier (including ECs, astrocytes, and choroid plexus), the blood-retinal barrier (in cell types such as retinal-pigmented epithelial cells and retinal Müller cells), and the blood-nerve barrier (in components like perineurium and endoneurial vascular endothelium) [23, 24]. Additionally, GLUT-1 is commonly present in various nephron segments, including podocytes, and it is predominant in glomerular mesangial cells [25]. This distribution of glucose transporters contributes to the understanding of why certain target tissues, such as the retina, kidney, and peripheral nerves are more susceptible to intracellular hyperglycaemia toxicity when blood glucose levels are not well regulated [11]. Consequently, these tissues are at a higher risk of developing microangiopathy, leading to prevalent diabetic microvascular complications, namely diabetic retinopathy, nephropathy, and neuropathy. These three conditions are collectively recognised as the classical targets of DM microvascular damage, and diabetic retinopathy, nephropathy, and neuropathy have been extensively studied and characterised as the most well-known diabetes-related microangiopathies. However, an intact microvascular bed is crucial for preserving every organ’s specific functions and achieving physiological balance to meet their respective metabolic demands. Therefore, microvascular dysfunction in DM can lead to widespread multiorgan consequences in non-classic target organs beyond the traditionally affected ones [1].

Despite sharing key physiopathological mechanisms that cause microvascular damage, the microcirculatory networks differ between organs due to variations in haemodynamic, vascular structure, and affected cells, resulting in diverse clinical presentations [20, 26]. This article reviews molecular mechanisms and pathological manifestations of diabetic microangiopathy in still-overlooked non-target organs, including the brain, the lung, the bone, the skin, the arterial wall, the heart, and the musculoskeletal system.

## Diabetes-related microvascular damage in the brain

### Structure and function of brain microcirculation

**Fig. 1:**
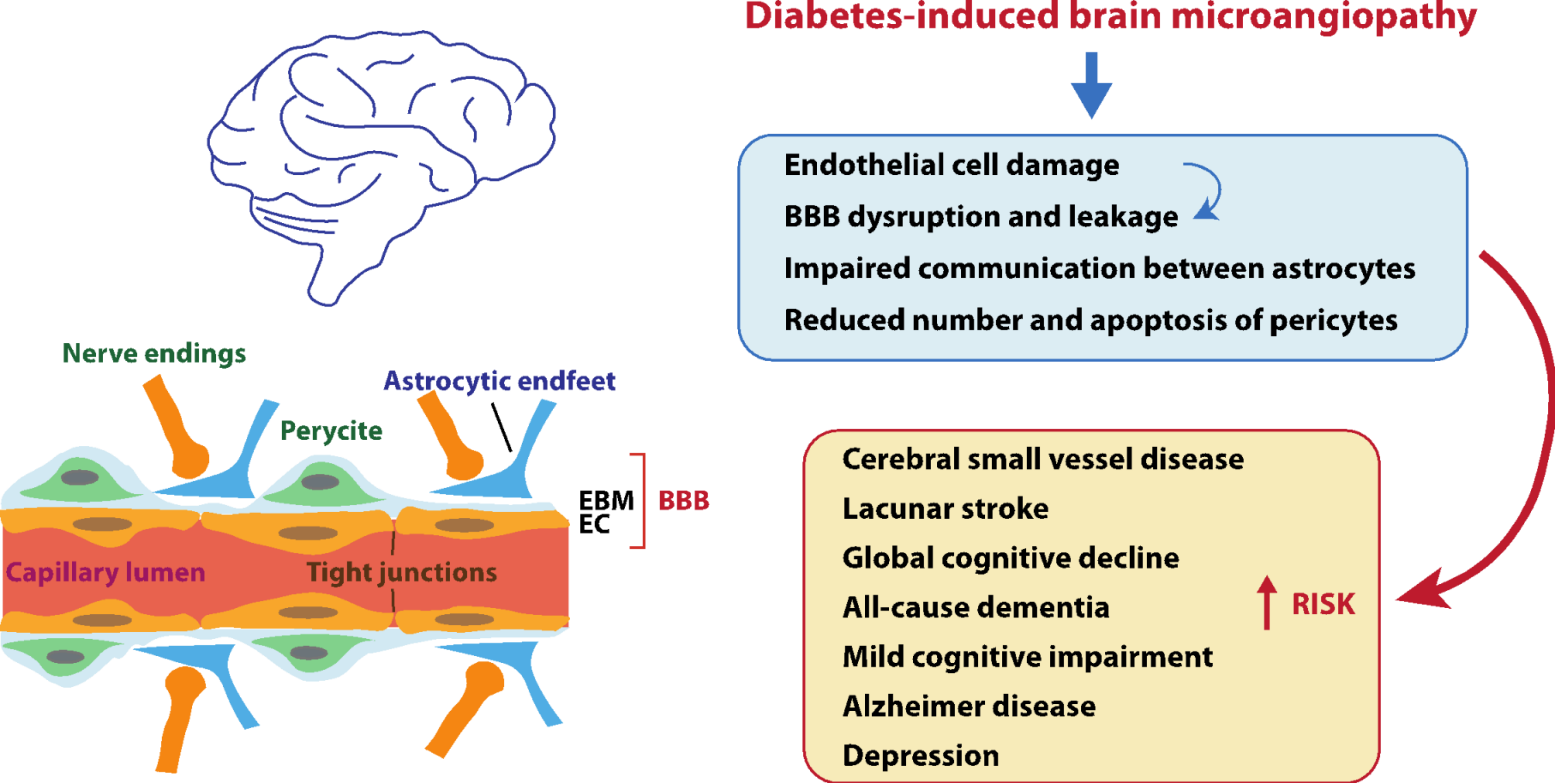
Schematic representation of the brain microcirculation, showing the neurovascular unit and the brain-blood-barrier (left); pathological findings and clinical conditions associated with brain microangiopathy observed in diabetes (right). BBB, blood-brain-barrier; EBM, endothelial basement membrane; EC, endothelial cell

The anatomy of the cerebral vasculature is inherently complex, as the brain’s metabolic demands must constantly adapt to its highly variable neuronal activity [27]. For this reason, flow changes are actively regulated at the arterioles and capillary levels to ensure an optimised local blood supply tightly coupled to neuronal activity [27]. This intricate coordination is achieved through a series of structural and metabolic collaborations between neurons, ECs, the endfeet of astrocytes, and pericytes, collectively forming the “neurovascular unit” (NVU) [27] (Fig. 1). Moreover, the ECs lining the cerebral vessels are closely interconnected through tight junctions that form the blood-brain diffusion barrier (BBB), separating the blood from the brain parenchyma (neurons and glial cells) [28]. The NVU closely interacts with the BBB and regulates its selective permeability, preventing the entry of most blood-borne agents into the parenchyma through paracellular routes [28].

### Pathological findings in brain microangiopathy in diabetes

Cerebral microvascular dysfunction can arise from almost any component within the NVU [29]. Glucose is the primary source of brain energy, entering the central nervous system (CNS) through glucose-transporter proteins and insulin receptors on the ECs of the BBB [30]. The existing evidence, predominantly derived from animal and in vitro studies, suggests that brain microvessels exposed to hyperglycaemic conditions can induce significant changes in the overall structure and function of the brain’s microcirculatory system through different, interrelated mechanisms [31], as detailed below (Fig. 1).

#### Endothelial cell damage and BBB leakage

ECs in cerebral vessels exhibit higher susceptibility to hyperglycaemia damage than cells in the brain parenchyma [32]. The BBB integrity in DM is compromised due to the loss of tight junction proteins, increasing the barrier’s permeability [33, 34]. While hyperglycaemia plays a significant role in BBB leakage in diabetes, other damaging factors, including diabetes-related hypertension, hyperlipidaemia, and insulin resistance, also contribute to the development of BBB dysfunction [31].

#### Impaired communication among astrocytes

Astrocyte endfeet around microvessels play a critical role in facilitating neurovascular communication [35]. In DM, swelling occurs in the astrocytic endfeet of microvessels, leading to the detachment of the plasma membrane from the basal lamina [36]. Consequently, communication between astrocytes via gap junctions is reduced, which hinders the astrocytic regulation of blood flow [31, 37].

#### Pericyte reduction and death

The retina and CNS exhibit the highest pericyte density [38]. Pericytes play a crucial role as essential effector cells in angiogenesis and microvascular remodelling processes, primarily through close interactions with the vascular BM [39]. Hyperglycaemia has been linked to reduced numbers and apoptosis of brain pericytes, resulting from oxidative stress triggered by the BBB disruption [40]. Moreover, reduced pericyte abundance is associated with increased vascular permeability, leading to haemorrhagic and hyperdilated vessels and potentially contributing to conditions like oedema [39]. Furthermore, pericyte death can result in the regression of newly formed microvessels, leading to fluid leakage, leukocyte adhesion to the vasculature, and hypoxia in the affected area [41].

### Clinical conditions associated with brain microangiopathy in diabetes

Microvascular brain dysfunction, called cerebral small vessel disease (CSVD), is the most common pathology underlying cognitive decline and vascular dementia worldwide [42, 43]. Magnetic resonance imaging (MRI) findings in CSVD include subcortical infarcts, lacunes, white matter hyperintensities (WMHs), enlarged perivascular spaces, cerebral microbleeds, cortical superficial siderosis, intracerebral haemorrhages, and brain atrophy [44, 45].

CSVD occurs naturally with ageing, but uncontrolled hypertension, DM, and hyperlipidaemia are among the leading risk factors [46]. In DM, there is an increased prevalence of CSVD features relative to controls (Fig. 1), which has been associated with the severity of diabetic retinopathy [47, 48]. Reported findings include a modest increase in WMHs [49], which is associated with poor glycemic control (high glycated haemoglobin, HbA1c) and glucose variability [50]. Additionally, T2DM is linked to a higher incidence with a 65% increased risk of lacunar strokes compared with those without diabetes [48, 51, 52]. Furthermore, a slight decrease in total brain parenchyma volume has been reported to be predicted by high HbA1c levels, long disease duration, and concomitant hypertension [51, 53]. Although controversial, some studies have also reported an increased risk of cerebral microbleeds [54–56]. Lastly, a meta-analysis of observational studies showed a significant association between prediabetes and an increased risk of infarcts or WMHs, while showing an inverse correlation with grey and white matter volume [57].

The clinical impact of CSVD in DM remains understudied, but the available evidence shows that these patients have a 25% increased risk of global cognitive decline and all-cause dementia, a 34% increased risk of mild cognitive impairment, a 43% increased risk of Alzheimer disease (AD), and nearly double risk of progression from mild cognitive impairment to dementia and vascular dementia when compared with subjects without DM [58]. Furthermore, subjects with prediabetes are also at higher risk of all-cause dementia and AD (18% and 36%, respectively) [58]. Lastly, high HbA1c levels, abnormal fasting plasma glucose, and high fasting plasma insulin levels have also been associated with dementia [58].

Beyond lacunar stroke and cognitive decline/dementia, clinical manifestations of CSVD in individuals with DM encompass a range of symptoms. Altered gait, such as reduced speed and poor performance when carrying out a competing task, is linked to white matter damage [59, 60]. Mood disturbances, including apathy and depression, are twice as common in T2DM as in the general population and are hypothesised to result from vascular injury in the frontal and subcortical brain regions [55, 61–63]. Lastly, two different meta-analyses have reported an increased risk of incident Parkinson’s disease in patients with diabetes [64, 65]. However, another systematic review and meta-analysis found inconclusive evidence [66], and a real-world study reported that the association was lost after adjusting for different risk factors such as duration of diabetes, smoking, body mass index, glycosylated haemoglobin, and comorbidities [67].

## Diabetes-related microvascular damage in the lung

### Structure and function of lung microcirculation

The lung is the only organ with two distinct circulatory systems: the pulmonary, which facilitates the exchange of gases between the bloodstream and the alveoli within the pulmonary capillary bed, and the bronchial circulation, which supplies oxygenated blood to the walls of the conducting airways, pulmonary arteries, and veins [68]. Besides effective gas exchange, the pulmonary vasculature also serves additional essential functions, including blood filtration to remove microemboli, participation in the metabolic regulation of vasoactive hormones, and the formation of a tight endothelial barrier that actively regulates paracellular extravasation of proteins, solutes and fluids to maintain interstitial fluid homeostasis [68].

### Pathological findings in lung microangiopathy in diabetes

The lung’s large vascular bed and the presence of abundant connective tissue make it an organ very sensitive to microvascular damage [69]. For instance, compared to capillaries in other organs and the pulmonary macrovasculature, pulmonary microvascular ECs generally exhibit much lower permeability and respond differently to barrier-disruptive stimuli such as pathogens, alterations in intracellular calcium, or oxidative stress [68].

**Fig. 2:**
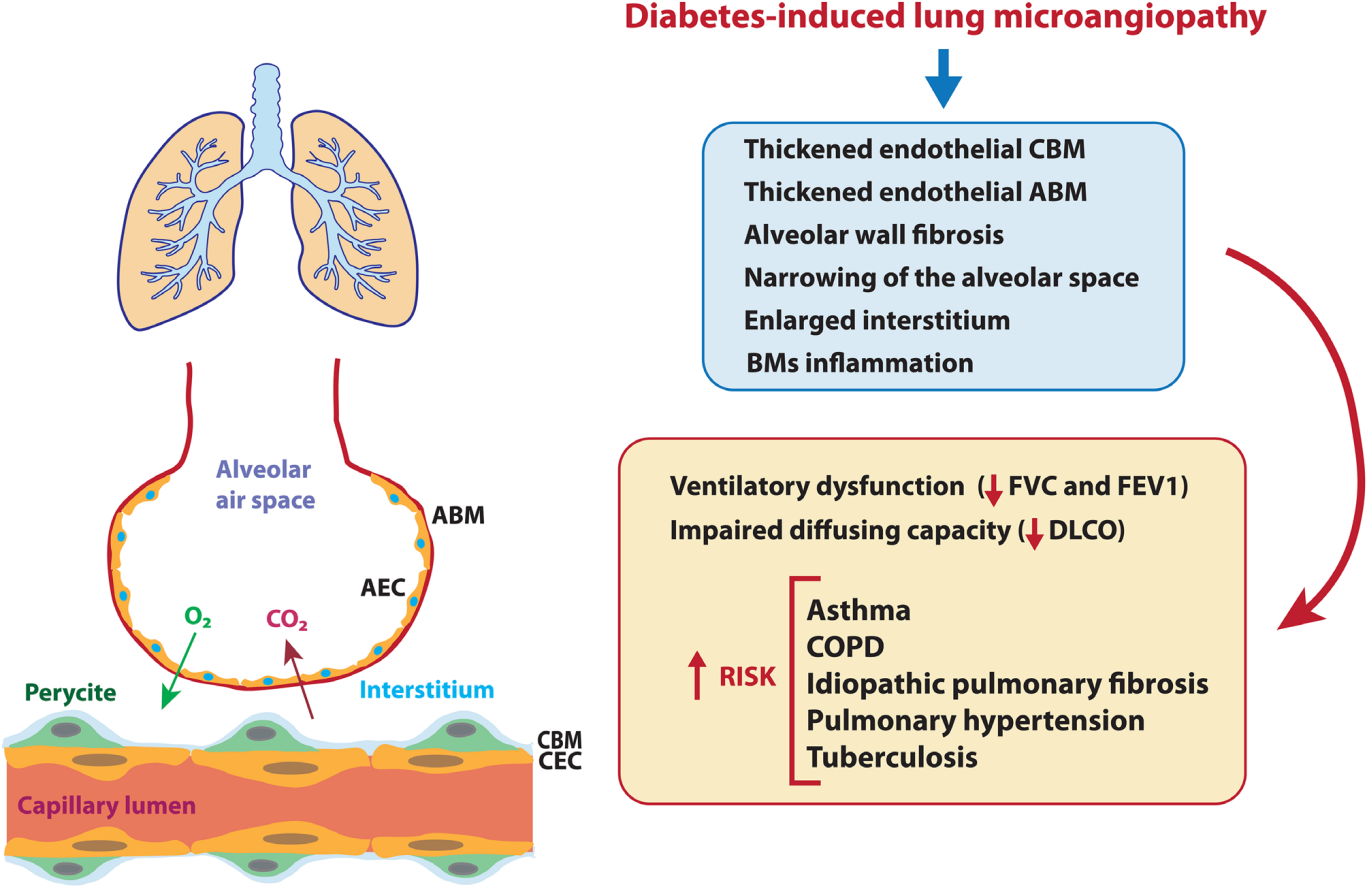
Schematic representation of the lung microcirculation, showing the blood-air barrier (left); pathological findings and clinical conditions associated with lung microangiopathy observed in diabetes (right). ABM, alveolar basement membrane; AEC, alveolar endothelial cell; CBM, capillary basement membrane; CEC, capillary endothelial cell; COPD, chronic obstructive pulmonary disease; DLCO, diffusing capacity of the lungs for carbon monoxide; FVC, forced vital capacity; FEV1, forced expiratory volume in the first second

The underlying pathogenic mechanisms of diabetes-lung injury are not fully understood [70, 71]. One proposed mechanism involves the combination of hyperglycaemia-induced microangiopathy and non-enzymatic glycosylation of connective tissue matrix proteins (elastin and collagen) [69]. Hyperglycaemia-induced microangiopathy can lead to thickening of the alveolar epithelium and basal lamina of pulmonary capillaries, resulting in reduced pulmonary capillary blood volume [69, 71–73] (Fig. 2). Furthermore, non-enzymatic glycosylation of connective tissue matrix proteins can lead to impaired vascular diffusion, stiffening of the lung parenchymal tissue, and reduced elasticity [69]. Overall, these changes impair the efficiency of gas exchange across the alveolar-capillary membrane and compromise the lung’s ability to expand and recoil during breathing, further contributing to respiratory difficulties [69, 73].

Morphological changes observed in post-mortem lung tissues of patients with DM compared to controls include thickened endothelial capillary basal lamina and alveolar epithelial cells and nodular fibrosis in the alveolar walls (Fig. 2) [74–77]. Besides, research in experimental animal models of diabetes has reported diverse structural modifications compared with control animals. These modifications include an increase in the proportion of alveoli per unit volume, the collapse of alveolar space because of increased fibrosis (with higher relative amounts of collagen, elastin, and basal laminae in the alveolar wall), narrowing of the alveolar space and enlarging the interstitium, as well as intense inflammatory reaction with neutrophil infiltration or aggregation in BM [78–82]. Additionally, biopsy specimens of bronchial mucosa of patients with diabetes have shown significantly thicker BM of bronchial epithelial cells compared to those without diabetes [83].

### Clinical conditions associated with pulmonary microangiopathy in diabetes

In clinical practice, diabetic lung injury manifests as ventilatory dysfunction and impaired pulmonary diffusing capacity (Fig. 2) [73]. Several meta-analyses and systematic reviews suggest that adult patients with either type 1 (T1DM) or T2DM show lower forced vital capacity (FVC) and forced expiratory volume in the first second (FEV1) than their non-diabetic counterparts [84–88]. This decrease in spirometry indexes is attributed to several factors, including the limitation of lung expansion due to an enlarged interstitium, decreased diffusion caused by the thickening and fibrotic changes of the alveolar-capillary basement membrane, and chronic low-grade tissue inflammation [84]. Moreover, meta-analyses and systematic reviews assessing the measurement of the diffusing capacity of the lungs for carbon monoxide (DLCO) show its reduction compared to patients without diabetes [85, 88]. Altered gas exchange in DM can be explained by the reduced alveolar-capillary membrane conductance and pulmonary capillary blood volume observed in these patients [73, 89]. Of note, the reduced lung function in diabetes is correlated with blood glucose levels, duration of diabetes and its severity, and is independent of sex, smoking status, or obesity [84, 87, 88, 90–92].

Lastly, it is worth mentioning that patients with DM are at increased risk of other pulmonary diseases, including asthma, idiopathic pulmonary fibrosis, chronic obstructive pulmonary disease (COPD), pulmonary hypertension, and infectious diseases such as community-acquired pneumonia, tuberculosis, and respiratory COVID-19 complications (Fig. 2**)** [70, 71, 93–97]. While the exact explanation for these comorbidities remains unclear, it has been suggested that many of these conditions share common mechanisms of endothelium damage-related lung injury with DM [70, 71].

## Diabetes-related microvascular damage in the bone tissue

### Structure and function of bone microcirculation

**Fig. 3:**
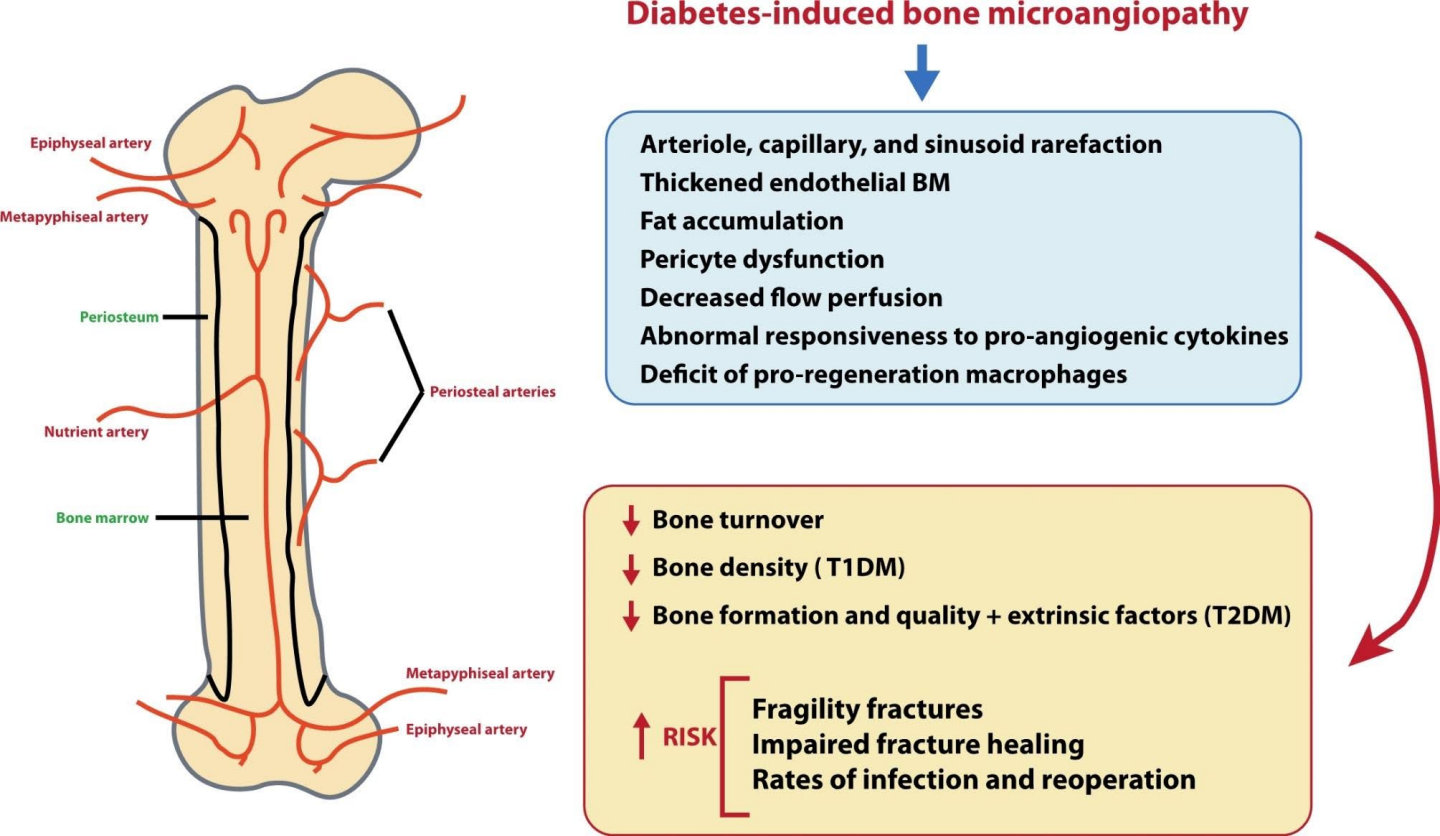
Schematic representation of the bone circulation (left); pathological findings and clinical conditions associated with bone microangiopathy observed in diabetes (right). BM, basement membrane; T1DM, type 1 diabetes mellitus; T2DM, type 2 diabetes mellitus

Bone is a highly vascularised and porous organ that receives blood supply from three systems: the nutrient artery, the metaphyseal-epiphyseal arteries, and the periosteal artery [98] (Fig. 3). The nutrient artery enters the diaphysis through the cortex into the medullar cavity and branches out to the metaphysis through arterioles and capillaries [99, 100]. Periosteal arteries supply the outer bone surface, with Haversian arteries running parallel to the longitudinal axis and shorter Volkmann’s arteries perpendicular to it. In contrast, in epiphyses and associated cartilage, arteries do not enter the medullary region but have a separate lateral blood circulation where arteries enter the bone from a heavy network of periarticular vascular plexus [99, 100].

In addition to the conventional role of delivering oxygen, nutrients, hormones, neurotransmitters, and growth factors to bone cells (osteocytes, osteoblasts, and osteoclasts), bone and marrow microcirculation plays a key regulatory role in bone development, remodelling, and repair [98, 101]. Moreover, it orchestrates the process of haematopoiesis and further contributes to bone mineralisation by delivering minerals such as calcium and phosphate to bone cells [98, 101].

### Pathological findings in bone microangiopathy in diabetes

The mechanisms of diabetic bone disease or diabetic osteopathy are still not fully revealed but are most likely multifactorial, with altered bone mineral density (BMD) through deposition of AGEs in the bone matrix, microarchitectural changes affecting bone strength, and altered bone turnover impairing bone remodelling processes [102, 103].

Despite the common underlying effect of glucose toxicity, the exact interplay of pathogenic factors and their contribution to diabetic bone disease varies between individuals with T1DM and T2DM [102–105]. In T1DM, reduced BMD results from deficient anabolic tone due to the lack of insulin, which plays a critical role in bone metabolism, along with high bone turnover [102–105]. Conversely, in patients with T2DM, who typically exhibit normal or increased BMD, pathogenic mechanisms of diabetic osteopathy are associated with insulin resistance, elevated levels of AGEs affecting collagen and bone matrix quality, and extrinsic factors such as increased mechanical loading due to obesity, or the impact of specific glucose-lowering medications such as insulin or thiazolidinediones [102–105].

Although some evidence exists regarding the effect of microangiopathy on diabetic osteopathy, the contribution of microvascular complications and the impairment of bone quality remains a subject of debate [105–107]. Both animal and human studies suggest that bone marrow microangiopathy in DM involves arteriole, capillary, and sinusoid rarefaction, BM thickening, and fat accumulation [108–110] (Fig. 3). Additionally, pericyte dysfunction [109], decreased flow perfusion [108], abnormal responsiveness to pro-angiogenic cytokines [111], and a deficit of pro-regeneration macrophages have been reported [112]. These findings align with the results of a meta-analysis showing an imbalance in bone metabolism and BMD in patients with DM and microvascular complications, as assessed by circulating bone turnover biomarkers [113]. The study found higher serum levels of P1NP (procollagen type 1 N-terminal propeptide, a marker of bone formation), parathyroid hormone (an indicator of calcium homeostasis), and CTX (C-terminal cross-linking telopeptide of type I collagen, an indicator of the rate of bone resorption and turnover), and osteocalcin (an indicator of bone turnover and bone metabolism), along with a decrease in serum 25(OH)D3 ( 25-hydroxyvitamin D3, indicator of bone mineralisation) [114]. Overall, bone microangiopathy in the context of DM appears to be linked to impaired bone metabolism balance, which may lead to bone loss or osteoporosis, and impaired fracture healing [107].

### Clinical conditions associated with bone microangiopathy in diabetes

The impaired bone health associated with DM predisposes patients to fragility fractures at most skeletal locations compared with the general population, along with an increased risk of impaired fracture healing (delayed union or non-union) linked to the absence of a proper vascular network in the fracture site [115, 116] (Fig. 3).

Multiple meta-analyses have consistently reported that both T1DM and T2DM are associated with a more than double increased risk of bone fractures, with patients with T1DM showing an excess risk more pronounced than those with T2DM [117–122]. This is surprising since low BMD is commonly observed in T1DM, whereas it is modestly increased or unchanged in patients with T2DM [123, 124]. This discrepancy is referred to as “the diabetic paradox of bone fragility”, wherein the increased fracture risk in T1DM is attributed to bone fragility, whereas in T2DM is associated with altered bone mechanical properties due to lower levels of bone turnover markers with reduced bone formation, and extrinsic factors such as obesity or deleterious drug effects [104, 121].

It is worth noting that a recent meta-analysis found a positive association between HbA1c levels and the risk of fractures at any site in both T1DM and T2DM [125]. The study reported that each 1% increase in the HbA1c level was linked to an 8% higher fracture risk [125]. Additionally, the research suggested that the elevated fracture risk associated with HbA1c levels might be partially explained by hypoglycaemia-induced falls, potentially triggered by insulin use [125]. Indeed, another meta-analysis on hip and non-vertebral fractures and a population-based matched cohort study found that the use of insulin was associated with an increased fracture risk among patients with T2DM, although this might also be related to hypoglycaemia as an adverse event associated with insulin therapy [122, 126].

Lastly, a recent meta-analysis found that patients with either T1DM or T2DM face a four-fold risk of increased risk of impaired fracture healing (including non-union, delayed union and malunion) in comparison with patients without DM [127]. Similarly, a systematic review found significantly increased rates of malunion in lower extremity fractures, along with increased rates of infection and reoperation, when compared with patients without diabetes [128]. Non-unions, which involve the disruption of the bone healing process, commonly occur due to the lack of a sufficient vascular network that supplies essential oxygen and nutrients to the fracture site [99].

## Diabetes-related microvascular damage in the skin

### Structure and function of skin microcirculation

Normal cutaneous microcirculation is organised in two parallel plexuses with capillary loops extending perpendicularly from the superficial plexus [129, 130]. Skin microcirculation controls temperature, tissue perfusion, capillary pressure and contributes to systemic blood pressure [129, 130].

### Pathological findings in skin microangiopathy in diabetes

The pathophysiology of skin complications in DM is complex and multifactorial, involving changes in microvascular and macrovascular circulation, decreased immunity, and altered collagen synthesis that collectively contribute to the development of skin complications [131]. Poor glycemic control and long disease duration are factors highly correlated with the occurrence of skin manifestations in DM patients [132, 133]. High blood glucose levels can directly disrupt corneocyte proliferation, keratinocyte and fibroblast functions and lead to vascular endothelial dysfunction and cell apoptosis [132, 133]. Moreover, they can indirectly cause skin complications through the formation of AGEs [132, 134, 135].

Skin capillarity density and recruitment (an increase in the overall number of perfused capillaries) have been found to correlate with insulin sensitivity and insulin-mediated vasodilatation in healthy subjects [136]. Moreover, systemic hyperinsulinemia exerts several effects on the skin, including the induction of capillary recruitment, increased NO-mediated vasodilatation, and enhanced microcirculatory blood flow [137]. Additionally, locally administered insulin results in a rapid increase in total skin blood flow, independent of systemic effects [137]. In subjects with T1D who do not exhibit clinical manifestations of diabetes-related complications, higher capillary density and recruitment have been observed using video-microscopy compared to matched controls [138]. Overall, these observations underscore the dynamic regulation of skin microcirculation by insulin.

Studies utilising nailfold capillaroscopy to examine the skin microcirculation network architecture have reported qualitative morphological changes in capillaries in patients with DM. In T1DM, increased capillary hydrostatic pressure and permeability are hypothesised to result from an adaptative basement membrane thickening [139, 140]. Other changes, more commonly observed in individuals with T2DM than in those without, include reduced capillary length, irregular distribution, tortuosity, avascular zones, ectasia, and abnormal morphology [56, 141–144]. Within the framework of the cross-sectional Maastricht studies, the assessment of dermal microcirculation through Laser Doppler flowmetry (LDF) found that the heat-induced skin hyperaemic response was lower in individuals with prediabetes and T2DM as compared with normoglycaemic individuals [145]. Importantly, this response was inversely correlated with HbA1c and fasting plasma glucose levels [145], further illustrating the impact of glycemic control on microvascular function. Additionally, laser speckle contrast imaging (LSCI) has revealed impaired endothelium-dependent skin microvascular vasodilator responses in patients with T1DM when compared to healthy subjects [146]. Lastly, a meta-analysis of studies assessing the local response to skin heating by LSCI or LDF reported that dermal microvascular function in patients with either T1DM or T2DM was largely reduced compared with healthy controls [147].

### Clinical conditions associated with skin microangiopathy in diabetes

Diabetes skin complications resulting from microvascular damage include several conditions, described in the following paragraphs.

***Diabetic dermopathy (DD)*** is the most typical skin condition affecting diabetes patients and consists of small, circumscribed, brownish atrophic skin lesions primarily found on the lower extremities [148]. Although the precise pathogenic mechanisms causing DD are not fully understood, the presence of microvasculopathy is the most widely accepted explanation [131, 149]. Notably, one of the significant indications of microangiopathy is the presence of thick-walled capillaries in the upper dermis [135].

***Diabetic thick skin*** encompasses two conditions: scleroderma-like skin and diabetic scleroderma. Scleroderma-like skin consists of painless, indurated, occasionally waxy-appearing, thickened skin [150]. On the other hand, diabetic scleroderma involves a gradual worsening of indurated and thickened skin [150]. The pathogenesis remains unclear, but both conditions have been associated with non-enzymatic glycosylation of connective tissue matrix proteins, leading to collagen deposition [150–152].

***Necrobiosis lipoidica*** is a rare chronic granulomatous disease that typically manifests as erythematous papules on the front of the lower extremities, which can coalesce to form atrophic telangiectatic plaques [150, 153]. The role of microangiopathy in this dermatosis remains controversial. However, glycoprotein deposition in the vasculature, leading to the thickening of blood vessels, ballooning degeneration of ECs of cutaneous capillaries, and localised proliferation of ECs invading the vascular lumen have been observed [135, 153].

***Diabetic blisters (bullosis diabeticorum)*** are uncommon noninflammatory eruptive blistering conditions that emerge in the acral regions in patients with a long duration of DM [135, 150]. The development of diabetic bullae is believed to involve various factors such as autoimmune processes, exposure to ultraviolet light, fluctuations in blood glucose levels, and neuropathy [150]. Additionally, changes in the skin microvasculature, characterised by thickened capillaries in the lesions, have also been implicated in this condition [150, 154].

***Periungual erythema*** refers to is erythema of the skin surrounding the nailbed. At the microangiopathy level, it is characterised by isolated homogeneous engorgement of venular limbs and simple capillary loops, sometimes accompanied by venous dilatation [135].

***Rubeosis facei***, or facial erythema, is a condition in which capillary microscopy has revealed venular dilatation linked to hyperglycaemia, predisposing to slow microcirculation in the cheeks of patients with diabetes [135].

***Pigmented purpuras*** are multiple brown to red macules that gradually merge into tan to orange patches on the legs, sometimes involving the ankles and dorsum of feet. These lesions result from red blood cell extravasation from the superficial vascular plexus, indicating abnormal capillary permeability [135].

***Erysipelas-like erythema*** is a well-demarcated lesion commonly found on the lower leg or the dorsum of the foot of diabetic patients indicative of functionally localised microangiopathy [135].

***Diabetic foot syndrome.*** The topic of diabetic foot syndrome, which includes foot ulcers as one of the skin lesions associated with diabetes, is presented in depth in a separate section of the manuscript.

## Diabetes-related microvascular damage in the arterial wall

### Structure and function of the arterial wall microcirculation

The inner layers of large arteries (tunica intima and tunica media) obtain their nutrients and oxygen directly through diffusion from the blood within their lumens [155, 156]. However, a supplementary network of microvessels known as the *‘vasa vasorum*’ (VV), derived from the Latin for ‘vessels of the vessels,‘ provides nourishment to the adventitial layer of these larger vessels [155–157]. The microvessels of the VV predominantly arise from branches of larger arteries and initially penetrate the artery wall through the adventitial layer before further entering the media layer (VV externa), although they may also originate from the luminal surface of the media (VV interna) and subsequently extend into the outer media [155–157].

*Vasa vasorum* are commonly present in the walls of many large arteries, including the aorta and its branches, carotid arteries, basilar and vertebral intracranial arteries, or pulmonary arteries [155–157]. The VV display a regularly layered vascular structure consisting of ECs, vascular smooth muscle cells, and surrounding connective tissue, implying their capacity to regulate their own tone in a manner similar to that of small coronary arteries [158].

### Pathological findings in *vasa vasorum* microangiopathy in diabetes

Changes in the equilibrium of vasoreactive factors and EC function within VV can lead to a functional decrease in blood flow to the vascular wall, resulting in local hypoxia and potentially triggering VV neovascularisation as a response to meet the arterial wall’s perfusion requirements [158]. Additionally, altered branching can occur due to processes such as atherosclerosis, where the vessel thickening creates a hypoxic environment that activates the angiogenic process in the adventitial VV [156].

Extensive research has investigated the role of VV in disease, particularly in the context of atherosclerosis, where VV microvascular dysfunction has been demonstrated to act not only as an initiator but also as an exacerbating mechanism in the susceptibility and progression of the disease [157]. Studies on the functional capabilities of the VV early in the course of hyperglycaemia are limited [155]. One study on post-mortem aorta arteries of T1DM patients showed thickened but disorganised VV in the artery walls compared to control subjects, possibly due to EC proliferation [159]. In addition, further studies have shown that patients with T1DM or T2DM without atherosclerotic disease have increased angiogenesis of the carotid VV compared with their nondiabetic counterparts [160–162]. Furthermore, a more recent study investigating post-mortem coronary arteries of patients with DM reported reduced VV density within both the adventitial and the entire vessel wall compared to individuals without DM or with prediabetes [163]. The study also found a negative correlation between higher HbA1c levels and VV density in the adventitia and the entire vessel wall [163]. Lastly, in lower atherosclerotic plaques of post-mortem thoracic and abdominal aorta, patients with DM showed increased severe inflammation, a significant increase in total neovessel content and mean neovessel density, as well as increased mean intra-plaque haemorrhage grade compared to plaques from non-diabetic patients [164].

### Clinical conditions associated with arterial wall microangiopathy in diabetes

The aforementioned mechanisms suggest that early VV dysfunction in DM may promote neovascularisation and contribute to atherosclerosis plaque formation, growth and progression, thereby adding to the increased risk of atherosclerotic CV disease observed in individuals with DM [155].

*Vasa vasorum* dysfunction has been investigated in animal models or human specimens of several cardiovascular diseases beyond atherosclerosis, where its role is well-established. Although still an area of ongoing research, VV loss has been suggested to contribute to the development of aortic aneurysms through wall weakening and aortic dissection as a result of microvascular tone loss; media VV leakiness; bleeding and hyperplasia of VV in the media of intracranial arteries have been implicated in influencing cerebral aneurysms growth, wall weakening, and an increased propensity for rupture; and hypoxia-induced pulmonary artery adventitial remodelling and neovascularisation have been reported in pulmonary hypertension (PH), potentially influencing the evolution of the disease [157].

Intriguingly, most epidemiological studies have reported that DM appears to be a protective factor for abdominal and thoracic aortic aneurysms (AAA and TAA, respectively), with DM patients less frequently experiencing AAA, small AAAs, and exhibiting slower AAA progression compared to non-DM patients [165]. However, while some factors, such as hyperglycaemia modulation of the extracellular matrix, might offer protection against AAA, the majority of vascular features associated with DM contribute, to some extent, to the development of artery aneurysms [166]. It is thus plausible that VV dysfunction may be among these contributing factors. However, it is also worth noting that certain glucose-lowering therapies, such as metformin, sodium-glucose co-transporter 2 (SGLT2) inhibitors, or incretin-based therapies, have demonstrated protective effects on the incidence and progression of AAA [166].

Regarding PH, epidemiological studies have shown that it is more frequent in patients with DM than those without DM, and the prevalence of insulin resistance is higher among subjects with PH than in the general population [167]. However, although diabetes-induced microvascular damage is thought to favour PH, the role of VV in this context has not been assessed.

## Microvascular heart damage in diabetes

### Structure and function of the coronary microcirculation

**Fig. 4:**
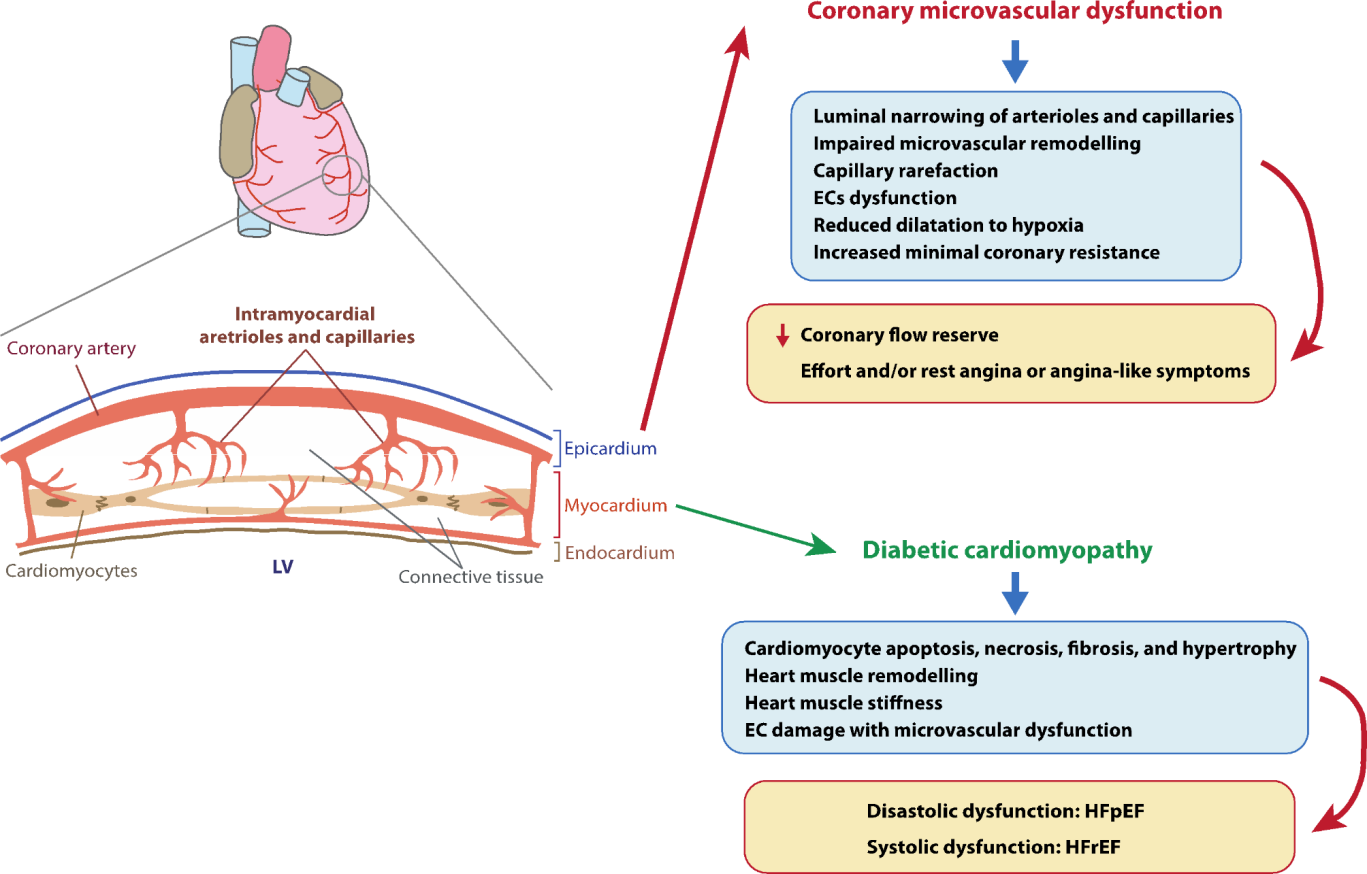
Schematic representation of the heart circulation (left) with a section showing the coronary arteries and the microcirculation in the myocardium; pathological findings and clinical conditions associated with heart microangiopathy observed in diabetes (right). EC, endothelial cell; HFpEF, heart failure (HF) with preserved left ventricular ejection fraction; HFpEF, heart failure (HF) with reduced left ventricular ejection fraction; LV, left ventricle

Coronary macrocirculation includes the larger blood vessels, namely coronary (epicardial) arteries, intramural arteries, and epicardial veins, while coronary microcirculation refers to the smaller vessels within the myocardium, including intramyocardial arterioles, capillaries, and venules (Fig. 4) [168, 169].

In contrast to other microcirculatory areas, the coronary microvasculature is continuously exposed to myocardial contraction. The primary function of coronary microcirculation is to regulate and maintain a constant coronary blood flow to ensure an adequate supply of oxygen and nutrients to the myocardium to meet its metabolic demands [170, 171]. At rest, approximately 75% of total coronary resistance resides in the small coronary arteries and arterioles [172]. Certainly, as myocardial oxygen consumption rises, myocardial metabolites synthesised by the endothelium (e.g., NO) prompt the dilation of metabolic-dependent arterioles and capillaries, leading to increased blood flow and local shear stress [171]. This, in turn, triggers the dilation of flow-rate-dependent vessels, such as arterioles, proximal precapillary arterioles, and epicardial arteries, further enhancing blood flow [171]. Besides, coronary vessels are highly adaptable, with acute modifications via the regulation of the vascular smooth muscle tone and chronic adaptations through changes in wall structure (e.g., structural remodelling and angiogenesis) [169, 170].

Diabetes-related microvascular dysfunction can lead to damage in both the coronary microvessels (coronary microvascular dysfunction; CMD) and the heart muscle itself (diabetic cardiomyopathy; DCM), each with its own distinct underlying pathophysiological mechanisms and clinical manifestations.

### Coronary microvascular dysfunction

#### Pathological findings in coronary microvascular dysfunction

The relationship between DM and CMD is not yet fully understood, but the pathological basis of CMD has been hypothesised to emerge from structural abnormalities (e.g., luminal narrowing of arterioles and capillaries, impaired microvascular remodelling, and capillary rarefaction) and/or associated functional abnormalities (e.g., ECs dysfunction and/or vasomotor smooth muscle dysfunction, leading to a blunted augmentation or reduction of coronary blood flow in response to stress) (Fig. 4) [168, 171, 173–175].

Animal models of diabetes have shown evidence of coronary microvascular dysfunction, manifested by reduced agonist- and flow-induced endothelium-dependent dilations of coronary arterioles, indicating impaired endothelium-dependent dilation in diabetes [176]. Morphological changes in arterioles of patients with DM include connective tissue accumulation and narrowing of the arterial wall, particularly in the most peripheral part, compared with their non-DM counterparts [177–179]. In both T1 and T2DM, there is an observable thickening of the arterial wall, reduced coronary flow reserve (CFR), increased minimal coronary resistance, and reduced dilatation to hypoxia compared to normal controls or patients without DM [180–183]. Moreover, studies using cardiac magnetic resonance (CMR) scanning have reported subclinical abnormalities of coronary vascular reactivity in T2MD patients [184]. Additionally, intracoronary Doppler studies have reported diminished coronary vascular reserve, and the endothelium-dependent coronary vasodilatation is impaired in DM patients with CMD compared to control subjects [185, 186].

#### Clinical conditions associated with diabetic coronary microangiopathy

Coronary microvascular dysfunction is defined as evidence of a reduced CFR despite the absence of obstructive epicardial disease and myocardial diseases [187, 188]. Symptoms related to supply-demand mismatch in coronary blood flow may manifest as effort and/or rest angina or angina-like symptoms (e.g., shortness of breath) [187, 188]. Unlike coronary epicardial atherosclerosis, CMD does not involve the development of atheroma. However, it is frequently associated with obstructive coronary artery disease (CAD) and cardiomyopathy [188, 189]. Notably, diabetes is a recognised cardiovascular risk factor for CMD [188, 189], affecting approximately 22% of patients with DM, according to a recent meta-analysis [190]. Although controversial, some studies have suggested a potential link between glycemic variability and control and reduced CFR or myocardial flow reserve in patients with DM [191–193].

#### Diabetic cardiomyopathy

The term diabetic cardiomyopathy (DCM) was initially coined to define a condition characterised by myocardial structural and functional abnormalities in the absence of CAD [194]. Over time, the definition has evolved to describe the increased susceptibility of people with DM to develop left ventricle (LV) dysfunction [195, 196]. Diastolic dysfunction is the hallmark of the disease and may lead to symptomatic heart failure (HF) with preserved left ventricular ejection fraction (HFpEF), a condition present in approximately 40–64% of asymptomatic patients with DM [197–199]. Over time, the disease may progress to systolic dysfunction with reduced ejection fraction (HFrEF or dilated phenotype) [199, 200].

#### Pathological findings in diabetic cardiomyopathy

Evidence on the underlying pathophysiological mechanisms of diabetic cardiomyopathy is derived from experimental, animal models, and data available from human studies, suggesting the involvement of several interrelated factors [195, 196, 198, 201]. These factors include glucotoxicity and lipotoxicity, systemic and cardiac insulin resistance, increased formation of AGEs, systemic and tissue low-grade inflammation, mitochondrial oxidative stress, cardiomyocyte autophagy, impaired calcium homeostasis, uncontrolled activation of the endoplasmic reticulum, dysregulation of the sympathetic nervous system, and microvascular dysfunction [195, 196, 198, 201]. The structural alterations observed in DCM are extensive and include cardiomyocyte apoptosis, necrosis, fibrosis, and hypertrophy, as well as cardiac remodelling, stiffness, and endothelial damage with microvascular dysfunction (Fig. 4) [195, 196, 198, 201].

#### Clinical conditions associated with diabetic cardiomyopathy

Over the past two decades, emerging evidence has highlighted the crucial role of coronary microvascular dysfunction in the development of HF in individuals with DM [195, 196]. Studies in patients with both DM and HF with HFpEF reported that they are at increased risk of hospitalisation for HF and cardiovascular death if they have concomitant microvascular complications (i.e., neuropathy, nephropathy, and retinopathy) [202, 203]. Furthermore, a recent observational study found that the risk of HF increased in parallel with the number of organs affected by microvascular disease in both T1 and T2DM [204]. Additionally, a study in human diabetic myocardial explants showed capillary rarefaction and pericyte loss, accompanied by reduced contractility and increased stiffness compared to nondiabetic explants [205]. The same study further investigated myocardial vascularisation in transgenic pigs and observed that hyperglycaemia induced capillary rarefaction in the myocardium, reduced ejection fraction, and impaired response to angiogenesis [205].

The classification of HFpEF and HFrEF as either a single phenotype representing successive stages of the disease or distinct phenotypes is a topic of ongoing debate [199, 201, 206, 207]. For instance, a study assessing patients with DM and microvascular complications found that HFpEF patients exhibited greater left ventricular remodelling and higher HF rates than those with HFrEF, suggesting that HFpEF might primarily manifest as a predominant microvascular disease [203]. At the pathophysiology level, specific mechanisms have been proposed for each phenotype [206–208]. In the preserved phenotype, left ventricular concentric remodelling is believed to result from coronary microvascular endothelial dysfunction triggered by a systemic inflammatory state, leading to cardiomyocyte hypertrophy and reactive interstitial fibrosis [206–208]. In contrast, the restrictive phenotype with eccentric left ventricular remodelling is thought to be driven by progressive cardiomyocyte cell death and extensive reactive replacement fibrosis due to ischemia-induced oxidative stress [206–208].

## Diabetes-related microvascular damage in the musculoskeletal system

### Structure and function of the musculoskeletal microcirculation

The musculoskeletal system is a complex and interconnected system that includes bones, tendons, muscles, ligaments, and joints. While ligaments and tendons have their own blood supply from the surrounding blood vessels (albeit some areas of tendons and ligaments remain avascular), bones are nourished from both intra and extra-articular blood vessels [209]. Skeletal muscle is highly vascularised, with the microvasculature serving a pivotal role in sustaining muscle contraction, which relies on the active consumption of energy substrates [210]. The microvascular unit branches from one or more feed arteries situated within the epimysium and extends into an interconnected network of arcade-like arterioles within the perimysium, subsequently infiltrating the endomysium in parallel with myofibers and ultimately giving rise to intricate capillary networks [210]. The microvasculature in skeletal muscle contains a continuous endothelial barrier equipped with tight junctions between ECs, which strategically restricts the passage of molecules, thereby limiting their access to the muscle tissue [211].

The components of the joint include the synovial membrane, besides bones, cartilage, tendons, and ligaments. Large arteries supply the fibrous capsule and synovium, establishing a nutritional connection with those in the periosteum and epiphysis, creating a cohesive nutritional unit supporting both the joint cavity and adjacent epiphysis [212]. In the joint capsule, blood vessels control the production of synovial fluid and maintain the articular cartilage [209]. The synovium is highly vascular to supply nutrients to the synovial fluid and the synovial membrane itself. It contains a terminal plexus composed of microvessels that form a series of finely anastomosing loops immediately under the synovial lining [212]. The synovium supplies nutrients to the synovial fluid and the membrane itself and can modulate inflammation and synoviocyte activity [209]. In contrast, articular cartilage remains avascular to maintain its mechanical performance, and it receives oxygen and nutrients by diffusion from the synovial microvasculature through synovial fluid or subchondral blood vessels [209].

### Pathological findings in the musculoskeletal system in diabetes

The pathogenic mechanism underlying the association between diabetes and musculoskeletal disorders remains elusive, but it is probably due to a combination of factors. Firstly, it is well established that hyperinsulinemia and hyperglycaemia are significant factors associated with bone metabolism damage. We refer the reader to the section on diabetes-related microvascular damage in the bone tissue for details. Additionally, inadequate glycaemic control and prolonged DM duration have been correlated with degenerative osteoarthritis in weight-bearing joints [213–215]. Secondly, the accumulation of AGEs in the BM leads to the loss of cartilaginous integrity on joint surfaces and deleterious structural changes in collagen cross-linkages [213–215]. These changes contribute to the development of osteoporotic and osteoarthritic conditions, as well as related issues like sarcopenia, tendinopathy, neuropathy, and joint stiffness [213–215]. Thirdly, diabetes-related chronic, low-grade inflammation is a key player in the progression of joint damage, stiffness, and pain [214, 215]. Lastly, impaired collagen synthesis and increased proteoglycan catabolism can alter the extracellular matrix composition, potentially leading to damage in bones, cartilage, and the surrounding periarticular connective tissues, including tendons, ligaments, joint capsule, and the synovial membrane [214].

### Clinical conditions associated with musculoskeletal microangiopathy in diabetes

Common musculoskeletal disorders observed in patients with DM include fibroproliferative disorders of the soft tissue (limited joint mobility [LJM], carpal tunnel syndrome, stenosing synovitis, Dupuytren’s contracture, and shoulder adhesive capsulitis), joint disorders (Charcot arthropathy and gouty arthritis), bone diseases (osteoporosis and fracture, previously discussed in another section of this review), and muscle disorders (diabetic sarcopenia and diabetic myonecrosis) [216]. The link between diabetes and these disorders is often supported by epidemiological studies that have documented a higher prevalence of these conditions among patients with DM compared to the general population, suggesting that DM is a potential risk factor (e.g., carpal tunnel syndrome, Dupuytren’s contracture, adhesive capsulitis of the shoulder, and gouty arthritis) [216, 217]. Besides, the involvement of microvascular damage has been frequently inferred from the fact that these DM patients commonly have underlying microvascular complications such as diabetic neuropathy, nephropathy, or retinopathy. Below, we only discuss musculoskeletal disorders that stem from either direct DM-related complications or share pathological mechanisms with microvascular disease, namely LJM, Charcot arthropathy, and diabetic myonecrosis.

***Limited joint mobility (LMJ)***, also known as diabetic cheiroarthropathy, is a long-term complication of DM characterised by progressive, painless stiffness of hands and fingers resulting in flexed contractures and hindered joint extension, accompanied by a thick, tight, and waxy skin texture prior to motion limitation [217, 218]. The prevalence of LJM in the general population ranges from 1 to 20%, whereas it occurs in 8–58% of patients with T1DM and 25–76% of patients with T2DM [213, 216, 217]. Moreover, it is more frequent among subjects with diabetes-related complications such as retinopathy, nephropathy, and neuropathy [219–221]. The underlying pathogenic mechanisms encompass genetic predisposition, intracellular hyperglycaemia-induced oxidative stress, and the formation of AGEs [217, 218, 222]. These factors collectively damage the endothelium, compromising vascular elasticity and inducing cross-linkages within proteins like collagen, which accumulate extensively within the skin [217, 218, 222].

An early histopathological study with hand’s skin biopsies of patients with T1DM and LJM found the following vascular changes: perivascular fibrosis of arterioles, which conferred them with a cord-like appearance penetrating deep into the dermal layers, an increase in vessel thickness, and reduced vascular lumen [223]. Another study assessed skin flow responses in patients with T1DM and LMJ compared to healthy controls [224]. The study reported reduced capillary flow in the T1DM patient’s palms in response to thermal challenge, suggesting a microvascular aetiology in the development of LMJ.

***Charcot arthropathy***, or diabetic neuroarthropathy, is a progressive degeneration of the foot and ankle joints induced by denervation [225]. This condition causes fractures and dislocations, leading to gross instability, deformity, and recurrent ulcerations, eventually leading to deformity when left untreated [225]. The prevalence of Charcot arthropathy is estimated at 0.1–0.4% in patients with DM, but this figure rises substantially to 35% among individuals with established diabetic peripheral neuropathy [226]. Regarding pathogenesis, different theories exist, not necessarily exclusive of one another. The neurotraumatic theory suggests that decreased protective proprioceptive mechanisms -secondary to neuropathy- predispose joints to damage and ligament relaxation, experiencing repetitive microtraumas that lead to complicated fractures and subsequent deformities during the healing process [217, 226]. The neuro-bone-inflammatory theory postulates an inflammatory response to repetitive microtrauma, possibly unnoticed because of reduced nociception, that initiates a vicious cycle of bone turnover and joint damage that persists due to uninterrupted weight-bearing [226, 227]. Lastly, the neurovascular hypothesis suggests that autonomic neuropathy triggers increased vasodilatation in the peripheral vasculature and prompts the formation of arteriovenous shunts, which redirect blood from the superficial capillary beds in the skin and channel it towards bones, leading to bone resorption and weakening, ultimately giving rise to fractures and deformities [226, 228].

A recent meta-analysis compared the foot cutaneous microvascular reactivity among three groups: patients with diabetes-related Charcot neuroarthropathy, patients with only DM or diabetic peripheral neuropathy (DPN), and healthy individuals [229]. The results showed that the cutaneous microvascular reactivity in people with Charcot neuroarthropathy was impaired compared to adults with uncomplicated DM, although less pronounced than this observed among those with DPN [229].

***Diabetic myonecrosis***, or diabetic muscle infarction (DMI), is a rare, spontaneous ischemic necrosis of skeletal muscle in the absence of arterial thromboembolism or an atherosclerotic occlusion of a large artery [213]. Typically, diabetic myonecrosis manifests as acute pain and swelling in one or a cluster of muscles within the thigh [230, 231]. The prevalence of this condition remains uncertain due to the predominance of case reports and limited case series in the available literature. However, it predominantly emerges in individuals with prolonged DM and inadequate glycemic control, and is often associated with microvascular complications -diabetic nephropathy, retinopathy, or neuropathy- in approximately 93% of cases [230, 231]. The pathophysiological mechanisms underlying DMI are yet to be fully elucidated, but it has been proposed that initial thromboembolic events, secondary to microvascular endothelial damage, trigger muscle ischemia [231–234]. This, in turn, sparks an inflammatory response accompanied by hyperaemia and reperfusion, leading to localised muscle damage and/or ischemic necrosis [231–234]. These findings are coupled with observations of hyalinised and thickened walls in small vessels, as well as narrowed lumens occluded by fibrin thrombi, causing vessel distention accompanied by an initial cellular response [233]. In older lesions, the lumens of small arteries are filled with loose connective tissue, in some cases recanalised [233].

Interestingly, a recent high-resolution electron microscope imaging study assessed the capillary fine structure in skeletal muscle biopsies from patients with T2DM compared with those of healthy subjects [235]. The findings showed several significant differences, including shorter intraluminal EC surface enlargement by projections, regarded as a capillary structural rearrangement, compared with controls [235]. Additionally, the pre-capillary BM was thickened, a greater number of capillaries showed disrupted BMs between pericytes and ECs, and a higher proportion of empty EC sockets (void of cell profile content) was evident among T2DM patients [235].

## Role of diabetes-related microvascular damage in diabetic foot and diabetic ulcers

The International Working Group defines diabetic foot (IWGDF) defines diabetic foot as “the presence of infection, ulceration, or destruction of tissues of the foot of a person with currently or previously diagnosed DM, usually accompanied by neuropathy and/or peripheral artery disease (PAD) in the lower extremity” [236]. Diabetic foot ulceration (DFU) is a serious complication of diabetic foot defined as a break of the skin of the foot that involves, as a minimum, the epidermis and part of the dermis [236]. The global prevalence of DFU in DM is 6.4%, between 50% and 60% of ulcers become infected, and 15% of DM patients require amputation [237].

In the pathological process that leads to diabetic foot, virtually all elements of the lower extremity come into play: skin, subcutaneous cellular tissue, muscles, bones, joints, vessels, and nerves [238]. The main pathological factors contributing to diabetic foot complications are peripheral vascular disease-induced ischemia, peripheral neuropathy leading to loss of protective sensation, and wound infection [239]. However, the co-existence of abnormal microcirculatory function has been shown to play a role in developing ulceration, gangrene, necrosis, and wound healing in diabetic foot [238, 240, 241]. The pathophysiological mechanisms underpinning neuropathy and PAD in the diabetic foot have been comprehensively covered by recent high-quality reviews and extend beyond the scope of the present discussion [237, 242]. Within this review, we are highlighting the available evidence regarding functional and structural impairment in microcirculation in the context of diabetic foot disease.

The most prominent structural microvascular abnormalities observed in post-mortem capillaries of skeletal muscle feet from DM patients include a thickened BM, pericyte degeneration, and the presence of acellular capillaries [243, 244]. Increased skin capillary BM thickness and enlarged capillary size have also been observed in foot skin biopsies of T1DM patients through light and electron microscopy [245]. Moreover, the width of the capillary BM appears to decrease in parallel with good glycemic control [246, 247]. In a more recent histological analysis examining capillary ultrastructure among T2DM patients, alongside the identified BM thickening, a reduction in capillary density within the dermal papillary layer was noted, together with a 58% decrease in the lumen area compared to patients without DM [241]. Moreover, the study found increased tunica media thickness due to smooth muscle cell proliferation, subsequently leading to occlusion of the arteriolar lumen [241].

Microvascular functional damage in the diabetic foot has been assessed by examining vasodilatory responses to stress or injury, which generally trigger heightened blood perfusion (reactive hyperaemia) in healthy microvessels to regulate blood supply locally [248]. In diabetic foot, available research has consistently shown a reduction in vasodilation response among individuals with DM in reaction to various stimuli. These include occlusion-induced responses (PORH) as well as thermally induced heating [240, 249, 250]. Multiple studies have reported the absence of transient vasodilation following localised pressure application (pressure-induced vasodilation; PIV) in patients with DM, with skin blood flow response diminishing at lower pressure levels compared to control subjects [240, 249, 250]. Overall, these alterations in microcirculatory function have been postulated to contribute to functional ischaemia and prompt the development of DFUs and delayed wound healing, the latter also related to endothelial dysfunction [240, 250–252].

The aforementioned changes apparently contradict the observed total increase in foot skin blood flow in FDU, giving rise to two different hypotheses to explain the underlying cause of the observed functional microvascular anomalies [249, 250]. One hypothesis, known as the haemodynamic theory, suggests that hyperglycaemia prompts an increase in microvascular flow and capillary pressure [249, 250]. This, in turn, triggers an endothelial injury response and, over time, microvascular sclerosis driven by the thickening of the capillary basement membrane due to long-term structural adaptation and remodelling. Ultimately, this cascade collectively results in limited response to local and central reflexes, such as reduced vasodilation and loss of reactive hyperaemia [249, 250]. The alternative hypothesis postulates that peripheral sympathetic denervation leads to increased peripheral blood flow and subsequent loss of vasoconstriction [249, 250]. This, in turn, would lead to increased blood flow through arteriovenous shunts, causing blood to be diverted away from the capillaries through these vessels (capillary steal), resulting in a reduced supply of oxygen and nutrients to the skin [249, 250].

## Conclusions and future perspectives

This review highlights that exploring and understanding diabetes microangiopathy requires awareness that microvascular complications represent a multi-organ disorder beyond traditional sites such as the retina, kidney, and peripheral nerves. As such, microcirculation impairment in commonly neglected target organs such as the brain, lung, bone tissue, skin, arterial wall, heart, or the musculoskeletal system, may lead to a broad spectrum of complications composing a constellation of clinical and subclinical signs and symptoms that is more likely than rare. Hence, it is crucial that healthcare professionals implement an expanded perspective to guide future investigations and interventions directed at alleviating the multifaceted manifestations of diabetes microangiopathy to improve patient care and outcomes.

## Data Availability

This article does not involve data sharing, as no datasets were generated or analysed in the course of the present study.

## Abbreviations

AAA: Abdominal aortic aneurysms
AD: Alzheimer disease
AGE: advanced glycation end products
BM: Basement membrane
BBB: Blood-brain diffusion barrier
BMD: Bone mineral density
CAD: Coronary artery disease
CFR: Coronary flow reserve
CMD: Coronary microvascular dysfunction
CMR: Cardiac magnetic resonance
CNS: Central nervous system
COPD: Chronic obstructive pulmonary disease
COVID-19: Coronavirus disease 2019
CSVD: Cerebral small vessel disease
DCM: Diabetic cardiomyopathy
DD: Diabetic dermopathy
DFU: Diabetic foot ulcer
DLCO: Diffusing capacity of the lungs for carbon monoxide
DM: Diabetes mellitus
DMI: Diabetic muscle infarction
DPN: Diabetic peripheral neuropathy
EC: Endothelial cell
FEV1: Forced expiratory volume in the first second
FDU: Foot disease ulcer
FVC: Forced vital capacity
HbA1c: Glycated haemoglobin
HF: Heart failure
HFrEF: Heart failure with restricted left ventricular ejection fraction
HFpEF: Heart failure with preserved left ventricular ejection fraction
LV: Left ventricle
LDF: Laser Doppler flowmetry
LJM: Limited joint mobility
LSCI: Laser speckle contrast imaging
MAPK: Ras–mitogen-activated protein kinase
MRI: Magnetic resonance imaging
NO: Nitric oxide
NVU: Neurovascular unit
PAD: Peripheral artery disease
PIV: Pressure-induced vasodilation
PH: Pulmonary hypertension
PKC: Protein kinase pathway
PORH: Post-occlusive reactive hyperaemia
RAGE: Advanced glycation end products (AGE) receptor
ROS: Reactive oxygen species
SGLT2: Sodium-glucose co-transporter 2
SVMC: Stimulating vascular smooth muscle cell
TAA: Thoracic aortic aneurysms
T1DM: Type 1 diabetes mellitus
T2DM: Type 2 diabetes mellitus
WMH: White matter intensity
VV: *Vasa vasorum*
